# Versican G3 Promotes Mouse Mammary Tumor Cell Growth, Migration, and Metastasis by Influencing EGF Receptor Signaling

**DOI:** 10.1371/journal.pone.0013828

**Published:** 2010-11-05

**Authors:** William Weidong Du, Burton B. Yang, Tatiana A. Shatseva, Bing L. Yang, Zhaoqun Deng, Sze Wan Shan, Daniel Y. Lee, Arun Seth, Albert J. Yee

**Affiliations:** 1 Department of Surgery, Sunnybrook Health Sciences Centre and Centre for the Study of Bone Metastasis, Odette Cancer Centre, University of Toronto, Toronto, Ontario, Canada; 2 Sunnybrook Research Institute, Department of Laboratory Medicine and Pathobiology, University of Toronto, Toronto, Ontario, Canada; University of Illinois at Chicago, United States of America

## Abstract

Increased versican expression in breast tumors is predictive of relapse and has negative impact on survival rates. The C-terminal G3 domain of versican influences local and systemic tumor invasiveness in pre-clinical murine models. However, the mechanism(s) by which G3 influences breast tumor growth and metastasis is not well characterized. Here we evaluated the expression of versican in mouse mammary tumor cell lines observing that 4T1 cells expressed highest levels while 66c14 cells expressed low levels. We exogenously expressed a G3 construct in 66c14 cells and analyzed its effects on cell proliferation, migration, cell cycle progression, and EGFR signaling. Experiments in a syngeneic orthotopic animal model demonstrated that G3 promoted tumor growth and systemic metastasis in vivo. Activation of pERK correlated with high levels of G3 expression. In vitro, G3 enhanced breast cancer cell proliferation and migration by up-regulating EGFR signaling, and enhanced cell motility through chemotactic mechanisms to bone stromal cells, which was prevented by inhibitor AG 1478. G3 expressing cells demonstrated increased CDK2 and GSK-3β (S9P) expression, which were related to cell growth. The activity of G3 on mouse mammary tumor cell growth, migration and its effect on spontaneous metastasis to bone in an orthotopic model was modulated by up-regulating the EGFR-mediated signaling pathway. Taken together, EGFR-signaling appears to be an important pathway in versican G3-mediated breast cancer tumor invasiveness and metastasis.

## Introduction

Versican is a member of the large aggregating chondroitin sulfate proteoglycans and is a member of the lecticans family [1]. To date, the entire primary structures of human [2], murine [3], bovine [4], and chick [5], versican coding mRNAs have been sequenced. Structurally all versican spliced forms include an N-terminal G1 domain, a glycosamingoglycan (GAG) attachment region, and a C-terminus containing a selectin-like (G3) domain [1],[6]. The G3 domain contains two epidermal growth factor (EGF)-like repeats, a lectin-like motif (carbohydrate recognition domain or CRD), and a complement binding protein (CBP)-motif. Versican interacts with its binding partners through its N- and C-terminal globular regions as well as its central GAG-binding region [7],[8]. By now four isoforms of versican (V0, V1, V2, V3) have been identified in various tissues [2]. The expression levels of versican V1 are high in the embryonic brain, wheras V2 is the dominant isoform with the mature central nervous system [4]. It is known to associate with a number of molecules in the extracellular matrix (ECM) including hyaluronan [9] tenascin-R [10], fibulin-1 and -2 [11] fibrillin-1 [12], fibronectin [13], P- and L-selectin, and chemokines [8]. Versican also binds to the cell surface proteins epidermal growth factor receptor (EGFR) [8], CD44 [14], integrin β1 [15], and P-selectin glycoprotein ligand-1 [14]. As a result, versican influences the regulation of cell motility, growth, and differentiation [16],[17],[18].

Given their ubiquitousness and high degree of conservation, it is likely that G1 and G3 play vital roles in proteoglycan function. The G1 domain has been shown to inhibit the attachment of glycosaminoglycan chains and the secretion of the products from the cell, while the G3 domain appears to enhance both processes [19]. There is increasing recognition of the importance of versican G1 to tumor growth, motility, and metastasis [20],[21]. Versican exerts its effect on astrocytoma cell migration and adhesion through the G1 domain [20]. In astrocytoma and chondrocyte morphogenesis, the effects of versican are greatly reduced when the G3 domain or EGF-like motifs within the G3 domain were deleted [22],[23]. Actually the terminal domains of versican may differentially control propagation of tumor cells and diversely modulate their responses to environmental hyaluronan [21].

Versican expression is enhanced during wound repair and tumor growth. In human tumors, versican is detected in the interstitial tissues at the invasive margins of breast carcinoma and in the elastic tissues associated with tumor invasion [22],[24],[25]. Immunolocalization of versican in breast tumors, including infiltrating ductal carcinoma, has been reported [26]. The high expression of versican in human breast tumors appears prognostic, being predictive of relapse, and negatively impacting overall survival rates [27],[28]. The mechanism(s) by which versican facilitates tumor growth and metastatic transformation in breast cancer is not well characterized. The phenotype of metastatic human breast cancer includes a predilection for spread to bony sites [29].

The EGF receptor is a transmembrane tyrosine kinase that is activated by tyrosine autophosphorylation after ligand-induced dimerization [30]. The Ras-Raf pathway leading to the activation of extracellular regulated protein kinases (ERKs) has been the subject of intense interest because of its role in the regulation of proliferation, differentiation, and cell–matrix interactions [31]. ERK1 and ERK2 are dually phosphorylated on threonine and tyrosine by the upstream MAP kinase, MEK. ERKs then phosphorylate and activate a variety of substrates including transcription factors, protein kinases and phosphotyrosine protein phosphatases leading to positive or negative regulation of signaling cascades [32].

Over-expression of EGF and its receptor (EGFR) has been found in many human tumors and cell lines, including breast cancer [33],[34]. With many other interactions occurring, the typical EGFR–MEK–ERK is believed to be a key element of a complex signaling network involved in survival signaling, cell migration, metastasis, and angiogenesis [35],[36]. Different approaches and targets are under evaluation and development for the therapeutic intervention of this key signal pathway in breast cancer [32],[37].

In the current study, we have investigated the mechanisms of versican G3 domain effects on mouse mammary tumor cell growth, migration, and proliferation. We also evaluated its effect on spontaneous metastasis to bone in an orthotopic model with focus on EGFR-mediated signaling. In order to investigate the effect of versican G3 on breast cancer cell growth and metastasis, we first characterized the expression of versican in murine mammary epithelial tumor cell lines 67NR, 66c14, 4T07, and 4T1, which were derived from a single spontaneous arising mammary tumor from Balb/cfC3H mice [38]. The tumorigenic and metastatic characteristics of these cells have been examined in detail [39],[40]. Although they share a common origin, these lines are phenotypically heterogeneous in their metastatic behavior. 4T1 cell line is one of the very few lines of any origin that spontaneously metastasizes to bone, closely mimicing Stage IV human breast cancer, which hematogeneously metastasizes to the lung, liver, bone, and brain via the hematogenous route, whereas 66c14 metastasizes to the lung and liver via the lymphatics [41]. 67NR cells fail to leave the primary site, while 4T07 cells are highly tumorigenic but fail to metastasize [39]. To evaluate the potential influence of versican G3 on EGFR signally, we exogenously expressed the versican G3 protein in a cell line with low basal versican, pEGFR, and pERK expression. Then we utilized a syngeneic orthotopic model of spontaneous breast cancer metastasis to test whether the exogenous expression of the versican G3 fragment in a mammary carcinoma 66cl4 cell line (a cell line that normally metastasizes to the lung but not to bone) was sufficient not only to promote local tumor growth but also to enhance metastasis to bone from the mammary fat pad.

## Materials and Methods

### Material Supplies

The monoclonal antibodies against ERK2, pERK, fibronectin, and CDK2, and the polyclonal antibodies against EGFR, pEGFR, cyclin A, cyclin B, cyclin D, cyclin E, and CDK6 were obtained from Santa Cruz Biotechnology. EGF, selective EGFR inhibitor AG 1478, selective MEK inhibitor PD 98059, hydroxyurea, and the monoclonal antibody against β-actin used in the study were obtained from Sigma. The polyclonal antibodies against versican V1 isoform, Glycogen synthase kinase-3β serine-9 phosphorylation (GSK-3β, S9P), and monoclonal antibody against vimentin were obtained from Abcam. The monoclonal antibodies against GSK-3β, N-cadherin, E-cadherin were obtained from BD Transduction Laboratories. Horseradish peroxidase-conjugated goat anti-mouse IgG and horseradish peroxidase-conjugated goat anti-rabbit IgG were obtained from Bio-Rad. Immunoblotting was performed using the ECL Western blot detection kit. Cell Proliferation Reagent WST-1, and High Pure PCR Template Preparation kits were obtained from Roche Applied Science.

### Versican expression in mammary tumor cell lines

Mouse mammary tumor cell lines 67NR, 66c14, 4T07, 4T1 were cultured in Dulbecco's Modified Eagle's medium (DMEM) supplemented with 10% fetal calf serum, penicillin (100 U/ml) and streptomycin (100 µg/ml) and maintained at 37°C in a humidified atmosphere of 5% CO2. Basal expression of versican amongst the four cell lines was compared by immunoblotting.

### Exogenous expression of versican G3 in breast cancer cell lines

The pcDNA1 - G3 construct and pcDNA1 - G3 fragment lacking the EGF-like motifs (G3ΔEGF) construct were generated by us [22],[23]. Mouse mammary tumor cell lines 66c14, 4T07, 4T1 and human breast cancer cell line MT-1 were transfected with pcDNA1-vecor and G3 constructs. Three days after transfection, Geneticin was added to the growth medium at a concentration of 1 mg/ml, and the cells were maintained in this medium until individual colonies were large enough for cloning. Chemically selected stable cell lines were maintained in medium containing 0.5 mg/ml Geneticin or stored in liquid nitrogen. The 66c14 cells were transiently transfected with G3 construct, G3ΔEGF construct, or the control vector. A leading sequence was engineered to both construct by us previously [19]. This leading peptide was obtained from link protein, which contains 180 nucleotides producing 60 amino acids. We have been using the system for many years and found that it is a powerful leading peptide for protein secretion. In addition, it contains an epitope recognized by the monoclonal antibody 4B6 [42].

### Cell attachment assays

Based upon experimental data demonstrating low basal expression of versican in 66c14 cells, a versican G3 construct was stably expressed in 66c14 cells using established techniques [22],[43]. The expression of versican G3 construct in the cell lysate and culture medium was examined with monoclonal antibody 4B6. Subsequently 2×10^5^ 66c14 cells transfected with versican G3 or control vector were seeded onto 6-well culture dishes in DMEM medium with varying amounts of FBS (2.5, 5, and 10%) for 3 h. Cell attachment assays were performed [19],[23]. Adherent cells were fixed, and the cell numbers were counted in randomly selected high power fields under an inverted light microscope. In selected experiments, cell suspensions were cultured with EGF (20 ng/ml), EGFR inhibitor AG 1478 (0.2, 2.0, and 5.0 µM), and selective MEK inhibitor PD 98059 (20 µM, 50 µM, 100 µM).

### Cell proliferation assays

Versican G3- and vector-transfected 66c14 cells (2×10^4^ cells) were seeded onto 6-well dishes in 10% FBS/DMEM medium and maintained at 37°C overnight. After 12–16 hours of culture, culture medium was removed and the cultures were washed with PBS, followed by culturing in DMEM with differing FBS concentrations (2.5, 5, and 10%). Cells were harvested daily and cell number was analyzed by coulter counter. Cell proliferation assays were also performed with colorimetric proliferation assay (Cell Proliferation Reagent WST-1). Versican G3 and control vector transfected 66c14 (2×10^3^ cells/well) cells were cultured in 100 µl FBS/DMEM medium in 96 wells tissue culture microplates. The absorbance of the samples against a background blank control was measured daily for 5 days by a microplate (ELISA) reader. In selected experiments, cell suspensions were cultured with EGF (20 ng/ml), EGFR inhibitor AG 1478 (0.2, 2.0, and 5.0 µM), and selective MEK inhibitor PD 98059 (20, 50, and 100 µM).

### Cell migration assays

#### (A) Wound-healing assay

Versican G3- and vector-transfected 66c14 cells (2×10^5^) were seeded onto 6-well dishes in 10% FBS/DMEM medium and maintained at 37°C until they reached 95% confluence. The monolayer G3- and vector-transfected cells were wounded by a sterile pipette tip to create a 1-mm cell-free path. Culture medium was removed and the samples were washed with PBS, followed by culturing in 10% FBS/DMEM medium with 2 mM of the cell growth suppressor hydroxyurea. Cells were fixed in 3.7% paraformaldehyde at the indicated time intervals (every 24 hours) and photographed under a low-magnification microscope. As well, the wounded cultures were incubated with medium containing 2.0 µM EGFR inhibitor AG 1478 or 50 µM selective MEK inhibitor PD 98059, followed by photography. The distances between the wounding centre and the front of the migrating cells (vertical axis) were measured for statistical analysis.

#### (B) Modified chemotactic Boyden chamber motility assays

This assay was performed using 24-well cell culture plates and a 3 µm cell culture insert. The tibias and femora were harvested from Balb/c mice, crushed and digested with a solution of DMEM containing collagenase type II (6 mg/ml) and dispase II (8 mg/ml) for 60 minutes. The cell suspension was filtered through a 70 µm nylon filter and washed three times by centrifugation in DMEM. The cell pellet was resuspended in DMEM, 10% FBS and maintained at 37°C overnight. After 12–16 h of culture, these cells were allowed to form a confluent monolayer in the bottom well of Transwell migration chambers. The medium was removed and the cultures were washed with PBS, followed by culturing in 600 µl 10% DMEM with or without 2.0 µM AG 1478, 50 µM PD 98059 at 37°C for an additional incubation of 2 hours. The G3-transfected 66c14 cells (1×10^5^ cells in 100 µl serum free DMEM medium with or without 2.0 µM AG 1478, 50 µM PD 98059) were gently injected into each filter insert (upper chamber) and then incubated at 37°C for 4 h. The filter inserts were removed from the chambers, fixed with methanol for 5 minutes, and stained with Harris' Haemotoxylin for 20 minutes. Samples were subsequently washed, dried, and mounted onto slides for analysis using a light microscope at 32 times magnification. Migrating cells were stained blue. Migration experiments were performed in triplicate and were counted in three fields of views/membrane.

### Western blot analysis

Protein samples were subjected to sodium dodecyl sulfate-polyacrylamide gel electrophoresis (SDS-PAGE) on separating gel containing 7–10% acrylamide. Separated proteins were transblotted onto a nitrocellulose membrane in 1× Tris/glycine buffer containing 20% methanol at 60 V for 2 hours in a cold room. The membrane was blocked in TBST (10 mM Tris-Cl, pH 8.0, 150 mM NaCl, 0.05% Tween 20) containing 5% non-fat dry milk powder (TBSTM) for 1 hour at room temperature, and then incubated with primary antibodies at 4°C overnight. The membranes were washed with TBST (3×30 minutes) and then incubated with appropriate horseradish peroxidase-conjugated secondary antibodies in TBSTM for 1 hour. After washing as above, the bound antibodies were visualized with an ECL detection kit as described previously [44].

### Cell cycle analysis

The expression of cell cycle-related proteins was analyzed by immumoblotting probed with appropriate antibodies as described above. The G3- and vector-transfected 66c14 cells were cultured in 10% FBS/DMEM media at 37°C, 5% CO_2_ with or without EGFR inhibitor AG 1478 (0.2, 2.0, and 5.0 µM), selective MEK inhibitor PD 98059 (20, 50, and 100 µM). The cells were washed and resuspended in cold PBS and incubated in ice-cold 70% ethanol for 3 hours. The cells were then centrifuged at 1,500 rpm for 10 minutes and resuspended in propidium iodide (PI) master mix (40 mg/ml PI and 100 mg/ml RNase in PBS) at a density of 5×105/ml and incubated at 37°C for 30 minutes before analysis with flow cytometry. Cell cycle related proteins cyclin A, cyclin B, cyclin D, cyclin E, CDK2, CDK6 and GSK-3β were analyzed by immunoblotting.

### In vivo tumorigenicity in balb/c mice, local tumor growth and metastasis

The G3- and vector-transfected 66c14 cells were cultured in 10% FBS/DMEM media at 37°C with 5% CO2. At 70% to 80% subconfluency, the cells were given fresh 10% FBS/DMEM media 24 hours before inoculation into the mice. Cell viability was determined by trypan blue exclusion, and cells were suspended with greater than 95% viability without cell clumping. Following appropriate institutional animal care committee approval, four-week-old Balb/c mice were injected transdermally with the G3- and vector-transfected 66c14 cells (1×10^7^ cells in 150 µl 10% FBS/DMEM medium) into the fourth (inguinal) mammary fat pad using a 1 ml syringe with a 26 G needle. Each group had 4 mice, which were chosen at random. Tumors were measured weekly thereafter. Four weeks after injection, animals were killed by CO_2_ inhalation for further analysis. At necroscopy, primary tumors, stromal tissues, lungs, liver, spine were dissected and kept frozen in liquid nitrogen for subsequent analysis. The vertebral spine was selected for evaluation of spread to bone given the predilection of bone metastasis to spread to this anatomic site.

### Tissue slide H&E staining, immunohistochemistry and immunoblotting

Primary tumors, lungs, spine, liver were also freshly excised and fixed in 10% formalin overnight, immersed in 70% ethanol, embedded in paraffin, and sectioned. The sections were followed by H&E staining and immunohistochemistry which were de-paraffinized with xylene and ethanol and then boiled in a pressure cooker. After washing with Tris-Buffered-Saline (TBS) containing 0.025% Triton X-100, the sections were blocked with 10% goat serum and incubated with primary antibody against versican G3 domain (4B6), or pERK in TBS containing 1% bovine serum albumin (BSA) overnight. The sections were washed and labeled with biotinylated secondary antibody, followed by avidin conjugated horseradish peroxidase provided by the Vectastain ABC kit (Vector, PK-4000). The slides were subsequently stained with Mayer's Hematoxylin for counter staining followed by slide mounting. For immunoblotting, the tumor primary tissues were grossly dissected into smaller pieces and lysated. The lysates were sonicated and cleared by centrifugation. The supernatant was subjected to SDS-PAGE and electroblotted onto the nitrocellulose membrane. After blocked with 5% milk/TBST for 1 hour, the membranes were incubated with monoclonal antibody against p-ERK and monoclonal antibody 4B6 (which recognizes an epitope at the C-terminal G3 domain) at 4°C overnight. After washing with TBST (3×30 minutes), the membranes were incubated with appropriate horseradish peroxidase-conjugated secondary antibodies in TBSTM for 1 hour. After washing as described, the bound antibodies were visualized with an ECL detection kit.

### PCR and Real-time PCR to measure tumor burden in the lung and bony spine tissues

Mouse lung and bony spine tissues were homogenized and the genomic DNAs were isolated with High Pure PCR Template Preparation kit according to the manufacturer's instructions. In order to estimate tumor burden, we extracted 3 samples from the above organs of each animal, and each sample was selected from 4 different positions in the organ. Tumor burden for each individual tissue was measured using PCR and q-RT-PCR incorporating Taqman chemistry. Primers and probes were designed using Primer Express, and were as follows: moVer7970F (5′ cgaggctagcggagctgttaccctttctccaact) and moVer10249R (5′ cgagctcgagcatgttcgccattttaaggatcag) for versican V1 isoform; CMVforward (5′ gtcatcgctattaccatggtgatgcgg) and CMVreverse (5′ agctctgcttatatagacctcccaccg) for genome typing;; β-actinforward (5′ ccggcatgtgcaaagccggcttcg) and β-actinreverse (5′ ctcattgtagaaggtgtggtgcc) for loading control. In regular PCR, genomic DNAs were processed in a PCR with two appropriated primers and the PCR products were analyzed on agarose gel and detected using ethidium bromide staining as described previously [45].

## Results

### Versican expression in mouse mammary tumor cell lines

**Figure 1:**
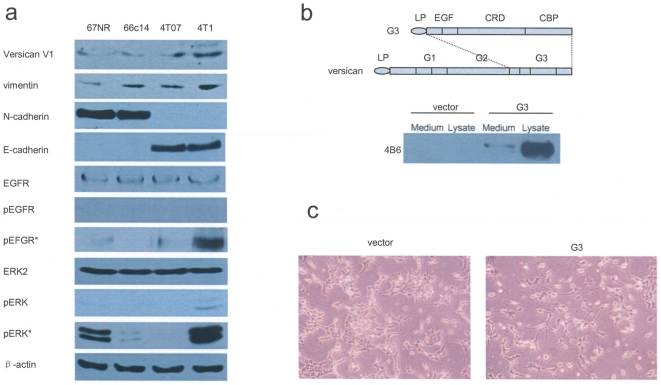
Structure of versican G3 domain and variable versican expression in mouse mammary tumor cells. (a) Immunoblotting showed that 4T1 cells expressed highest level of versican V1 isoform, vimentin, pERK; 67NR and 66c14 cells expressed N-cadherin, while 4T07 and 4T1 cells expressed E-cadherin; In 20 ng/ml EGF medium, 4T1 cells expressed increased pEGFR and pERK. EGFR*: adding 20 ng/ml EGF for 5 min; ERK*: adding 20 ng/ml EGF for 60 minutes. (b) Versican G3 construct (upper) was expressed in 66c14 cells, analyzed by western blot using cell lysate and culture medium (lower). (c) Morphologically, the G3-transfected 66c14 cells appeared more elongated and spreading more evenly as compared with the predominantly cuboid appearance of cells in the control group that tended to aggregate into clusters.

We have previously demonstrated that versican plays important roles in mediating cell activities [46],[47]. To understand how versican modulates signaling pathways associated with tumor metastasis, we examined expression of versican V1 isoform (250–300 kDa) and the related molecules in different cell lines known to possess different capacities in tumor metastasis. Though RT-PCR showed that there was not much difference of versican V1 expression in mRNA level among the 4 cell lines (Fig. S1a), versican V1 protein expressed differently in the four mouse mammary tumor cell lines. It is highly expressed in 4T1 cells, and expressed in low levels in 4T07 and 66c14 cells. Derived from a single spontaneously arising mammary tumor from a Balb/C mouse, these 4 mouse mammry tumor cell lines show the same expression of versican V1 in mRNA level. However, translational controlling and modification may play roles in differential expression of versican V1 protein in these 4 cell lines. 4T1 cells also expressed the highest level of vimentin and pERK. The expression of EGFR and ERK2 in the 4 cell lines was similar. 67NR and 66c14 cells expressed N-cadherin, while 4T07 and 4T1 cells expressed E-cadherin. When treated by 20 ng/ml EGF for 5 minutes, 4T1 cells expressed the highest level of p-EGFR. When 4T1 cells were treated by 20 ng/ml EGF for 60 minutes increased pERK expression was observed (Fig. 1a). To investigate the effect of versican G3 on breast cancer cell growth and metastasis, and its potential signaling pathways, we exogenously expressed a versican G3 construct in 66c14 cells (Fig. 1b). The expression of versican G3 in cell lysate and culture media of 66c14 transfected cells when compared with vector control cells is also depicted in Figure 1b. Morphologically, the G3-transfected 66c14 cells appeared more elongated and spread more evenly in vitro as compared with the predominant cuboid appearance of cells that tended to aggregate into groups in the vector control group (Fig. 1c).

### Versican G3 enhances breast cancer cell adhesion

**Figure 2:**
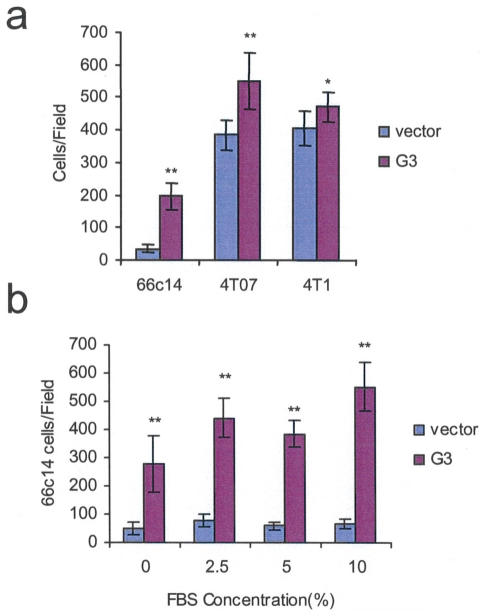
Versican G3 domain enhances cell attachment. (a) G3- and vector-transfected 66c14, 4T07, and 4T1 cells (2×10^5^) were inoculated in 6-well culture dishes in DMEM containing 2.5% FBS for 2 hr. More G3-transfected cells attached on the dishes than the vector control ones. (b) The G3- and vector-transfected 66c14 cells (2×10^5^) were inoculated in 6-well culture dishes in DMEM containing 0, 2.5, 5, and 10% FBS; more G3 transfected cells attached after 3 hours. (*n* = 9, * p<0.05, ** p<0.01, analyzed with *t*-test).

In the cell attachment assays, G3- and vector-transfected 66c14 cells, 4T07 cells, and 4T1 cells were inoculated in 6-well culture dishes. After the cells were incubated in 2.5% FBS/DMEM medium for 2 hours, we observed enhanced cell attachment to culture dishes in the G3 group as compared with the vector control (Fig. 2a). Cultured in 2.5, 5, and 10% FBS/DMEM medium for 3 hours, we observed that more G3-transfected 66c14 cells attached to the dishes (Fig. 2b). Blockade of EGFR with AG 1478, or treating the cells with selective MEK inhibitor PD 98059 did not influence G3-induced cell attachment during the time period evaluated (Fig. S2a, Fig. S2b).

### Versican G3 activates the EGFR/ERK pathway

**Figure 3:**
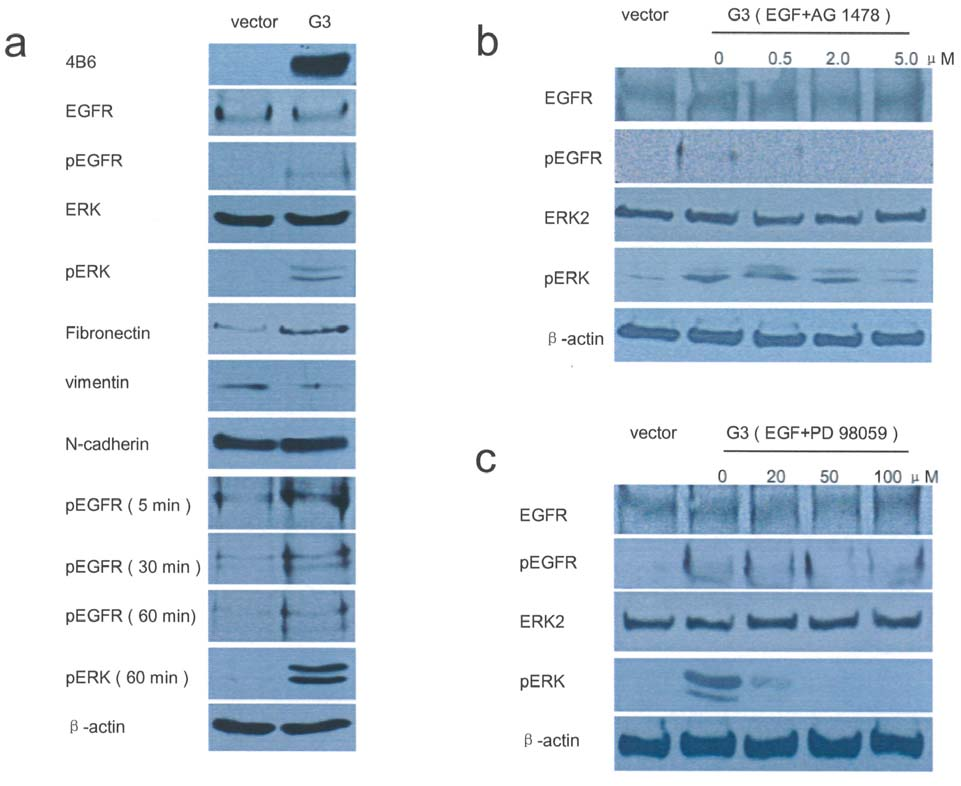
pEGFR and pERK activity is enhanced by versican G3 domain. (a) Immunoblotting showed that the G3 construct did not alter the total EGFR and ERK proteins but dramatically increased the levels of pEGFR and pERK. G3 up-regulated expression of fibronectin and down-regulated vimentin expression. Treated with EGF (20 ng/ml), the G3-transfected 66c14 cells expressed increased levels of pEGFR and pERK. (b) Treated with EGF (20 ng/ml) and different concentrations of selective EGFR inhibitor AG 1478 (0.2, 2.0, and 5.0 µM) increased expression of pEGFR and pERK, which could be blocked by the selective EGFR inhibitor AG 1478. (c) Treated with EGF (20 ng/ml) and different concentrations of selective MEK inhibitor PD 98059 (20, 50, and 100 µM), PD 98059 blocked G3-induced upregulation of pERK but not of EGFR.

Immunoblotting showed that expression of G3 construct in 66c14 cells did not alter the total proteins of EGFR, ERK2, and N-cadherin, but dramatically increased the levels of pEGFR and pERK. The presence of G3 also up-regulated fibronectin expression and down-regulated vimentin expression (Fig. 3a). Cultured in 20 ng/ml EGF medium for 5-60 minutes, the G3-transfected cells expressed increased levels of pEGFR and pERK (Fig. 3a). Treated with 20 ng/ml EGF and different concentrations of selective EGFR antagonist AG 1478 (0.2, 2.0, and 5.0 µM), the G3-activated pEGFR could be blocked with increased dose of the inhibitory agents (Fig. 3b). Expression of pERK was also inhibited in the G3 expressing cells cultured in the medium with 5.0 µM AG 1478. Treated with 20 ng/ml EGF and different concentrations of selective MEK (ERK kinase) inhibitor PD 98059 (20, 50, and 100 µM), G3-induced expression of pERK, but not of pEGFR, could be blocked by PD 98059 (Fig. 3c).

### Versican G3 expression enhances breast cancer cell proliferation in 66c14 cells via up-regulating the EGFR/ERK signaling pathway

**Figure 4:**
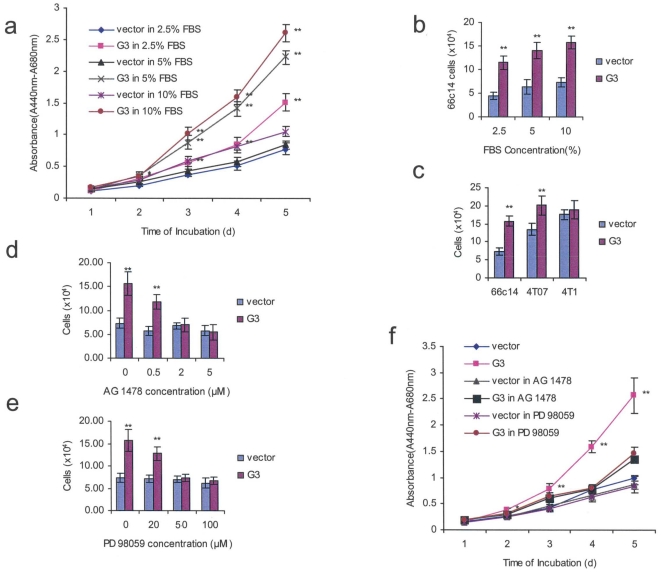
Expression of versican G3 enhances cell proliferation by upregulating the EGFR/ERK pathway. (a) The G3- and vector-transfected 66c14 cells (1×10^3^) were inoculated in 96-well culture dishes and cultured in DMEM medium containing 2.5, 5, and 10% FBS for 5 days. Proliferation assays performed with colorimetric proliferation assay indicated that the G3 construct enhanced cell growth. (All groups compared with vector control cells, *n* = 8, * p<0.05, ** p<0.01, analyzed with *t*-test) (b) The G3- and vector-transfected 66c14 cells (2×10^4^) were inoculated in 6-well culture dishes containing 10% FBS/DMEM and cultured for 3 days. The G3-transfected cells grew faster as compared with the control group. (c) The G3- and vector-transfected 66c14, 4T07, and 4T1 cells (2×10^4^ cells) were inoculated in 6-well culture dishes with 10% FBS/DMEM and cultured for 3 days. The G3-transfected cells grew faster in these cell lines as compared with the control group. (All groups compared with vector control cells, *n* = 6, * p<0.05, ** p<0.01, analyzed with *t*-test). (d) 2×10^4^ G3- and vector-transfected 66c14 cells were inoculated in 6-well culture dishes and treated with AG 1478 (0.5, 2.0, and 5.0 µM) cultured for 3 d. Analysis by light microscopy revealed that treatment with dose of 2.0 or 5.0 µM AG 1478 prevented G3 induced cell proliferation. (e) The G3- and vector-transfected 66c14 cells were treated with PD 98059 (20, 40, and 100 µM) and cultured for 3 days. Treatment with 40 or 100 µM PD 98059 prevented G3 induced cell proliferation. (f) Cell growth assays showed AG 1478 (2.0 µM) and PD 98059(50 µM) blocked G3 enhanced cell growth. (All groups compared with vector control cells, *n* = 8, * p<0.05, ** p<0.01, analyzed with *t*-test).

Versican G3 expression not only enhanced tumor cell adhesion, but also enhanced cell proliferation in different culture conditions using DMEM medium with varying concentrations of FBS. Cell proliferation assays were performed, which indicated that the G3 construct enhanced cell growth in DMEM medium containing 2.5, 5, and 10% FBS when cultured for over 5 days (Fig. 4a). To confirm these results, G3- and vector-transfected 66c14 cells were inoculated in 6-well culture dishes in 10% FBS/DMEM medium. After the cells were cultured for 12 h, the medium was changed to contain different concentrations of FBS (2.5, 5, and 10%), and the cells were cultured for an additional period of 3 days. Greater cell viability was observed in the G3 group as compared with the control group (Fig. 4b, 4c, and Fig. S3b).

Inhibitors were used to test whether versican G3 activated breast cancer cell proliferation through EGFR-mediated signaling. G3- and vector-transfected 66c14 cells were treated with 0.5, 2.0, or 5.0 µM of EGFR inhibitor AG 1478 for 3 days. Analysis by light microscopy revealed that treatment with the dose of 2.0 or 5.0 µM AG 1478 prevented G3 induced cell proliferation (Fig. 4d). We also cultured G3- and vector-transfected 66c14 cells (2×10^4^) in 10% FBS/DMEM with selective MEK inhibitor PD 98059 (20, 50, and 100 µM) for 3 days. Treatment with the dose of 50 or 100 µM PD 98059 inhibited G3-induced proliferation (Fig. 4e). Cell growth assays performed with colorimetric proliferation assay showed that both AG 1478 (2.0 µM) and PD 98059 (50 µM) blocked G3-enhanced cell growth (Fig. 4f). These results suggest that versican G3 domain promoted breast cancer cell growth through activating EGFR/ERK pathway; blockade of EGFR or ERK prevented G3 induced enhanced breast cancer cell proliferation.

### Versican G3 domain promotes cell cycle entry through EGFR/ERK signaling and expression of CDK2 and Glycogen synthase kinase-3β serine-9 phosphorylation (GSK-3β, S9P)

**Figure 5:**
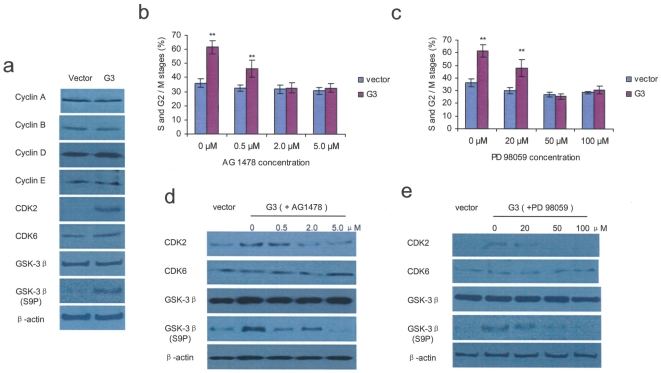
Versican G3 domain promotes cell cycle entry through the EGFR/ERK pathway. (a) The expression of cell cycle-related proteins was analyzed by immumoblotting probed with appropriate antibodies as indicated in the methods. β-actin expression was used as a loading control. Expression of cyclin A, cyclin B, cyclin D, cyclin E, CDK6, and GSK-3 β was similar in G3- and vector control cells. G3 expressing cells recorded higher levels of CDK2 and GSK-3 β (S9P) compared with vector control cells. (b) 5×10^4^ G3- and vector-transfected 66c14 cells were inoculated in 6 well culture dishes and cultured in 10% FBS/DMEM without or with AG 1478 (0.5, 2.0, and 5.0 µM) for 2 days. Flow cytometry results showed that more G3 expressing cells (62%) were in S, G2 and M stages than the vector cells (36%). Treating with 2.0 µM or 5.0 µM AG 1478 prevented G3 enhanced S, G2 and M cell cycle status. (c) Similarly, culturing the cells with different concentrations of selective MEK inhibitor PD 98059 (20, 50, and 100 µM), treated with 50 or 100 µM PD 98059 prevented G3 induced enhancement of S, G2 and M cell cycle status. (d) Immunoblotting showed that selective EGFR inhibitor AG 1478 blocked G3 induced expression of CDK2 and GSK-3 β (S9P). (e) Immunoblotting confirmed that selective MEK inhibitor PD 98059 blocked G3 induced expression of CDK2 and GSK-3 β (S9P). (All groups compared with vector control cells, *n* = 6, * p<0.05, ** p<0.01, analyzed with *t*-test).

To estimate the effect of G3 on the cell cycle, we tested expression of cell cycle-related proteins by immunoblotting using methods as described [48], [49]. Expression of cyclin A, cyclin B, cyclin D, cyclin E, CDK6, and GSK-3β was similar in G3- and vector-transfected cells, while G3 expressing cells maintained high levels of CDK2 and GSK-3β (S9P) (Fig. 5a). Experiments with flow cytometry indicated that more G3 expressing cells (62%) were in S, G2 and M stage as compared with the vector-transfected cells (36%). Treatment with 2.0–5.0 µM AG 1478 or 50–100 µM PD 98059 inhibited the G3-induced proportional increase of cells in S, G2 and M stages, the effect being dose-related (Fig. 5b and 5c, Fig. S4 and S5). Immunobloting showed that 2.0–5.0 µM selective EGFR inhibitor AG 1478 blocked G3 induced expression of CDK2 and above 5.0 µM AG 1478 also blocked G3 enhanced expression of GSK-3β (S9P) (Fig. 5d). While selective MEK inhibitor PD 98059 prevented G3 promoted expression of CDK2 with concentration of 20–100 µM, and blocked G3 induced expression of GSK-3β (S9P) at 50–100 µM (Fig. 5e).

### Versican G3 enhances breast cancer cell motility through EGFR-mediated signaling

**Figure 6:**
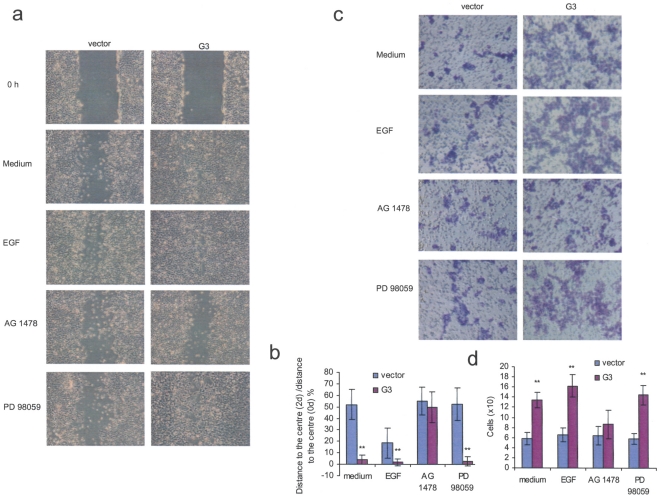
Versican G3 enhances tumor cell motility through EGFR signaling. (a) The G3- and vector-transfected 66c14 cells (2×10^5^) were inoculated in 6-well culture dishes and cultured for 12 h. Monolayer G3- and vector-transfected cells were wounded by a sterile pipette tip to create a 1-mm cell-free path, washed with PBS, and then cultured in 10% FBS/DMEM medium with 2 mM hydroxyurea. These samples were also treated with or without 20 ng/ml EGF, 2.0 µM 1478PD, 50 µM PD 98059. G3-transfected cells exhibited enhanced migratory capacity, which was prevented by EGFR inhibitor AG 1478, but not by PD 98059. (b) The distances between the wounding centre and the front of the migrating cells (vertical axis) were measured for statistical analysis. (All groups compared with vector control cells, *n* = 10, * p<0.05, ** p<0.01, analyzed with *t*-test) (c) The chemotactic motility assays were performed using 24-well cell culture plates and the 3.0 µm cell culture insert. The results from a representative experiment are shown. (d) The G3-transfected 66c14 cells showed enhanced migratory capacity toward the mouse bone stromal cells that was also prevented by EGFR inhibitor AG 1478, but not by PD 98059. The modified chemotactic Boyden chamber motility assays were performed four times and were counted in three fields of views/membrane.

In wound healing assays, G3-transfected cells exhibited enhanced migratory capacity to the wounding areas, as compared with the vector control cells (Fig. 6a, Fig. 6b, Fig. S3b). However, G3 enhanced tumor cell migration to the wounding areas was significantly inhibited by EGFR antagonist AG 1478 but not by MEK inhibitor PD 98059 (Fig. 6a, Fig. 6b), suggesting that versican G3 enhanced breast cancer cell motility through EGFR signaling in a mechanism that did not involve the ERK downstream pathway. Using the modified chemotactic Boyden chamber motility assays, versican G3-transfected 66c14 cells showed enhanced migratory capacity toward the mouse bone stromal cells, which was also prevented by EGFR inhibitor AG 1478, but not by MEK inhibitor PD 98059 (Figs. 6c and 6d).

### Versican G3 domain promotes tumor growth and spontaneous metastasis in the orthotopic model

**Figure 7:**
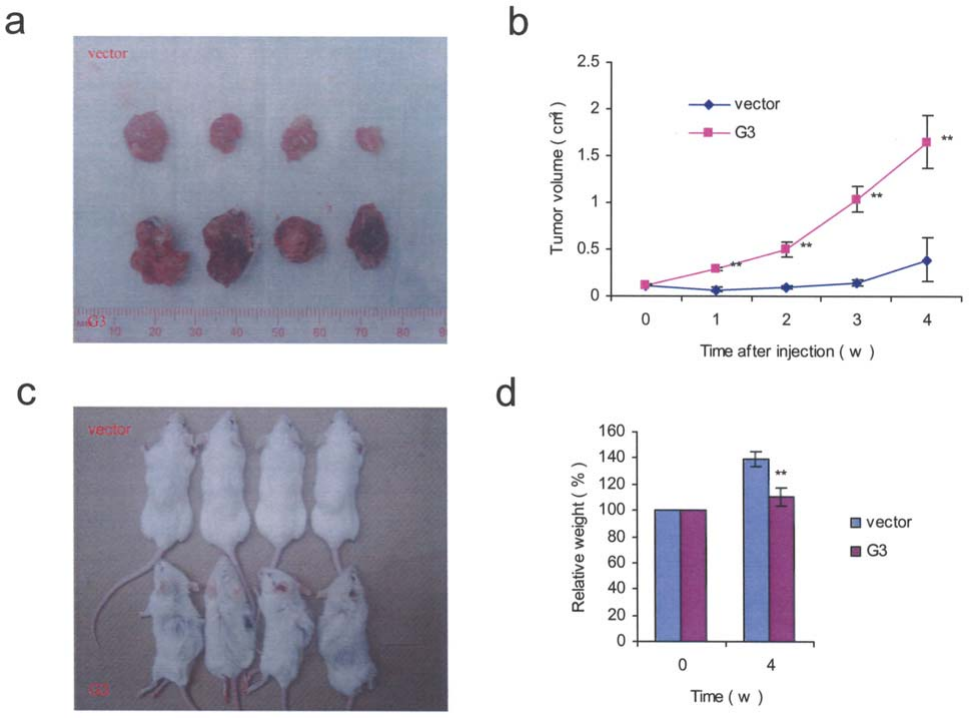
Versican G3 domain promotes tumor growth and systemic metastasis in an orthotopic model. (a) Animals treated with G3-transfected cells grew larger tumors compared with the control group (p<0.05). (b) Tumor growth curve demonstrated that G3 treated tumor grew faster than that of the control group (*n* = 4, *p<0.05, **p<0.01, analyzed with t-test). (c) Balb/c mice inoculated with the G3-transfected 66c14 cells appeared cachetic after 4 w. (d) At necroscopy, a more significant weight loss pattern was observed in the G3-treated group (*n* = 4, *p<0.05, **p<0.01, analyzed with *t*-test).

**Figure 8:**
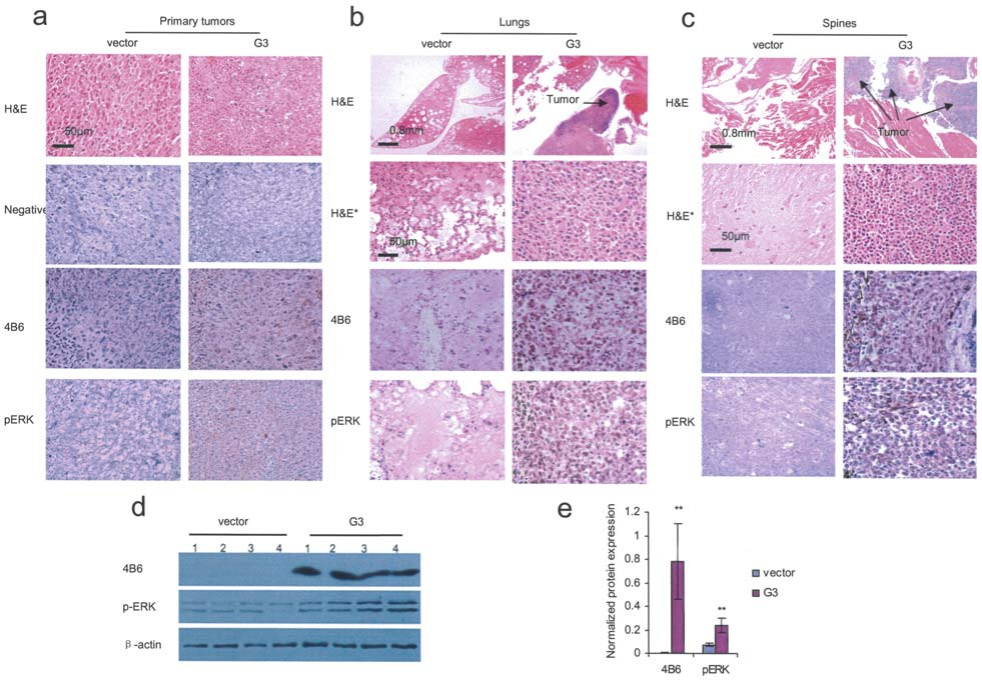
Versican G3 and pERK are expressed at high levels in both primary and metastatic tumors arising from G3-transfected cell inoculations. (a) Immunohistchemistry staining showed that both versican G3 and pERK expressed at high levels in primary tumors arising from G3 transfected cell inoculations. (b) H&E of the versican G3 expressing 66c14 cells inoculated spinal metastasis lesions, which express high levels of 4B6 and pERK in immunohistchemistry staining. (c) H&E of the G3 expressing 66c14 cells inoculated lung metastasis lesions, expressing high levels of 4B6 and pERK in immunohistochemistry staining. (d) Immunobloting showed that versican G3 and pERK were expressed at high levels in the G3-transfected cell treated tumor. (e) Expression of versican G3 and pERK were visualized by chemi-luminescence, quantified by Alphaimager 3400 and normalized to β-actin protein controls (*n* = 12, **p<0.01, G3 versus vector,).

Balb/c mice were inoculated by transdermal injection in the dorsal paraspinal fat pad with G3- or vector-transfected cells. Each group had 4 mice, which were assigned to experimental groups randomly. All the other mice were sacrificed 4 weeks after treatment. At necroscopy, animals treated with the G3-transfected cells produced larger tumors as compared with the control group (*p*<0.05) (Figs. 7a and 7b). Balb/c mice inoculated with G3-transfected cells became cachectic after 4 weeks (Fig. 7c). A more progressive weight loss pattern was also observed in the G3 group (p<0.05) (Fig. 7d). Tumor growth kinetics demonstrated that the G3 treated tumors grew faster than that of the control group (*p*<0.05) (Fig. 7b). All of the animals in the versican G3 group (4/4) developed lung metastasis when compared to 25% (1/4) in the control group (*p*<0.05). To test whether versican G3 expression enhanced EGFR/ERK signaling pathway in vivo, paraffin sections of primary tumor, lung, and spine were stained with H&E and immunohistochemistry stained with anti-pERK and and anti-G3 (4B6) antibodies. The experiments demonstrated that both versican G3 and pERK were stained at high levels in the primary tumors arising from the G3-transfected cells (Fig. 8a). Mice in the versican G3 group developed metastatic lesions in lung and spine, which also expressed high levels of pERK and 4B6 (Figs. 8b and 8c). Tumor tissues of G3- and vector-expression cell treated mice were digested and lysated. Immunoblotting indicated that versican G3 and p-ERK were expressed at high levels in tumors arising from the G3-transfected cell inoculations when compared with the controls (Figs. 8d and 8e).

**Figure 9:**
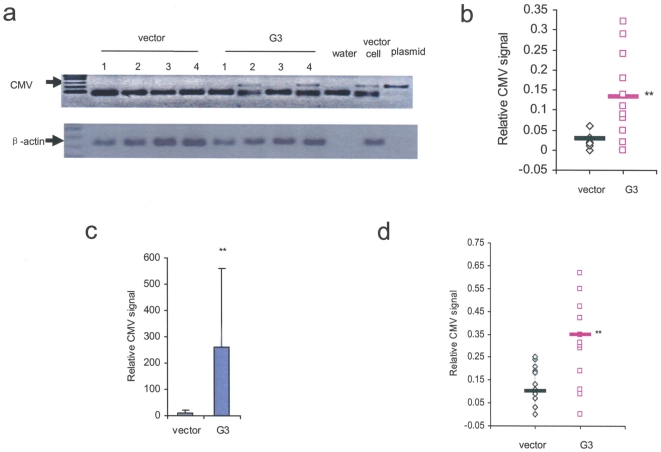
Versican G3 domain promotes breast cancer cell metastasis in vivo. (a) The CMV signal was not detected in the spine tissues of the vector control mice, but could be detected in the G3-treated group. (b) Expression of CMV signal was visualized and quantified by Alphaimager 3400 and normalized to β-actin signal controls. Each point represents one sample, but not all points are distinguishable. The average normalized CMV signal for each group is shown with a line (*n* = 12, *p<0.05, analyzed with *t*-test). (c) The tumor burden in spine was measured by real-time quantitative PCR as described in Materials and Methods. The metastatic tumor burden of spine increased 25-fold in the G3-treated mice than in vector control group (*n* = 12, *p<0.05, analyzed with *t*-test). (d) The metastatic tumor tissues in mouse lungs were detected by PCR as described in the study methods.

Tumor burden in the bony spine was detected by PCR and real-time quantitative PCR as described [50]. The CMV signal (CMV sequence present in stably integrated vector in tumor cells only) was not detected in the spine tissues of the vector control mice (0/4), but observed in those of the G3 treated group (2/4)(Fig. 9a). CMV signal was higher in the spine tissues of G3 treated animals than those of the vector control group (*p*<0.05) (Fig. 9b). Real-time PCR demonstrated that the relative metastatic tumor burden in the spine increased 25-fold over 4 weeks in G3 treated mice than in the vector control group (*p*<0.01) (Fig. 9c). The PCR results also confirmed that the metastatic tumor burden in the lung was much higher in the G3 treated group than in the vector control group (*p*<0.05) (Fig. 9d).

### Versican G3 domain promoted tumor cell growth and migration are related to its EGF-like motifs

**Figure 10:**
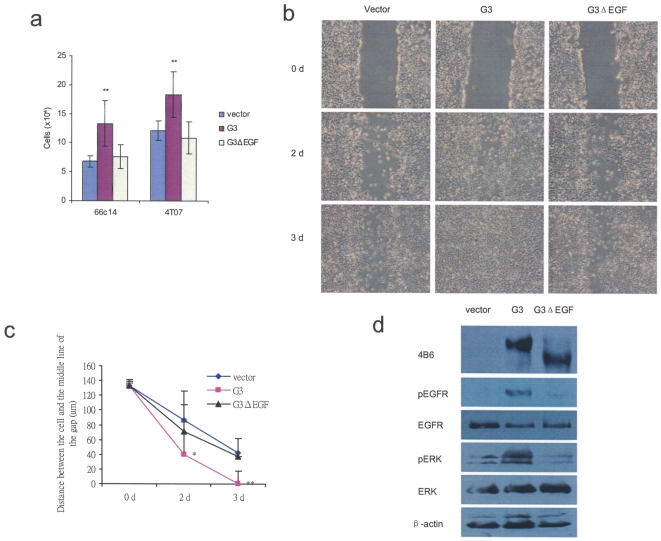
Versican G3 domain modulates breast cancer cell growth and migration via its epidermal growth factor-like motifs. (a) Vector-, G3- and G3ΔEGF- transiently transfected 66c14 cells and 4T07 (2×10^4^) were inoculated in 6-well culture dishes containing 10% FBS/DMEM and cultured for 3 days. Cells were counted under light microscopy. (b) Vector-, G3- and G3ΔEGF- transiently transfected 66c14 cells (2×10^5^) were inoculated and cultured in 6-well culture dishes containing 10% FBS/DMEM for 12 h. all the samples were wounded by a sterile pipette tip to create a 1-mm cell-free path, washed with PBS, cultured in 10% FBS/DMEM medium with 2 mM hydroxyurea for 3 d. (c) The distances between the wounding centre and the front of the migrating cells (vertical axis) were measured for statistical analysis. (All groups compared with vector control cells, *n* = 10, * p<0.05, ** p<0.01, analyzed with *t*-test). (d) Cell lysates of vector-, G3- and G3ΔEGF- transiently transfected 66c14 cells were prepared and subjected to immunoblotting with antibodies to 4B6, pEGFR, EGFR, pERK and ERK2.

The key functions of the EGF-like motifs of versican G3 domain were well demonstrated by our former study [22],[23]. Here we transiently transfected cells with G3 construct, G3 fragment lacking the EGF-like motifs (G3ΔEGF), and the vector, and found that G3ΔEGF expression did not show enhanced cell growth and migration as G3 transfected cells did (Fig. 10a, 10b, 10c). Immunoblots showed that G3ΔEGF expressing cells did not show enhanced pEGFR and pERK as G3 transfected cells did (Fig. 10d).

## Discussion

Interaction of versican with the extracellular matrix (ECM) and cell surface proteins is believed to enhance structural integrity between tumor and stromal tissues and regulates cell proliferation and metastatic potential. Versican's effect on proliferation may be related to its C-terminal G3 domain [22]. In astrocytoma, versican G3 enhances tumor growth by interactions with β1 integrin and angiogenic factor VEGF [46]. Versican/PG-M G3 domain appears to be important in local and systemic tumor invasiveness of human breast cancer and may enhance connectivity between tumor cells and surrounding stromal components, in addition to facilitating neo-vascularization through interactions with VEGF and fibronectin [47]. Versican G3 enhances cell proliferation in NIH3T3 fibroblasts. This effect is mediated, in part, by the action of versican EGF-like motifs on endogenous EGF receptors [51]. Previous studies have demonstrated that versican G3 enhances neurite growth by enhancing the epidermal growth factor receptor (EGFR), which is associated with activation of EGFR-mediated signaling through G3's EGF-like motifs [52]. In this study we demonstrated that G3 enhances mouse mammary tumor cell growth, migration, proliferation and metastasis through up-regulating EGFR signaling.

Given the frequency at which abnormalities in EGFR signaling are present in human breast cancer and observations of how these changes influence tumor cell survival, migration, metastasis, and angiogenesis, EGFR has been an attractive target for therapeutic manipulation. The presence of two EGF-like domains in versican G3 and the importance of versican as a prognostic factor in breast cancer add to the interest in further delineating the role of EGFR and downstream signaling in invasive breast cancer [47].

Versican G3 domain appears to be important in local and systemic invasiveness of human breast cancer [47]. The mechanism behind G3 induced tumor invasiveness was of interest in the present study. Our study demonstrated that over-expression of versican G3 in mammary cell lines with low basal versican expression (ie. 66c14 cells) enhanced mammary cancer growth through up-regulating active EGFR expression and activating the EGFR/ERK pathway. Enhanced metastasis that included bony sites such as the spine also appeared mediated in part through EGFR signaling. We have demonstrated that versican G3 domain appreciably increased breast cancer cell attachment, proliferation, and migration in vitro, and promoted local tumor growth and metastasis in vivo. Both selective EGFR inhibitor AG 1478 and selective MEK inhibitor PD 98059 could block this signaling pathway and prevent versican G3 induced effects on mammary cancer cell proliferation. Versican G3 expression also enhanced mammary cancer cell motility by EGFR-mediated signaling. As selective EGFR inhibitor AG 1478 blocked G3 effects on tumor cell migration while MEK inhibitor PD 98059 did not suggest that ERK was the main downstream signaling component when specifically considering effects on cell migration. Significant G3 effects on the cell cycle were also observed. G3 construct promotes cell cycle entry by expressing CDK2 and GSK-3β (S9P). Blockade of the EGFR/ERK pathway prevents G3 induced expression of CDK2 and GSK-3β (S9P) and as a result blocks cell cycle entry.

Recent advances in the mechanisms of oncogenesis have revealed a close relationship between the cell cycle and apoptosis. The progression of a cell through the cell cycle is promoted by cyclin dependent kinases (CDKs), which are positively regulated by cyclins and negatively regulated by CDK inhibitors [53],[54]. In progressively growing tumors, constitutive activation of the EGFR/ERK pathway allows for G0-G1-S-phase transition and cell division [55]. High levels of p38 or p27 activity are believed to be a negative growth regulator and may suppress cell proliferation by inhibiting ERK, inducing G0-G1 arrest, triggering senescence or apoptosis [56],[57]. Any effectors that alter the balance of p27 and CDK2, ERK and p38 may have profound consequences for tumor growth and survival. Our study demonstrates that versican G3 domain activates cell cycle entry and growth by dramatically increasing expression of pERK, CDK2, which alters the balance of p27 and CDK2, and ERK and p38. In addition, both selective EGFR inhibitor AG 1478 and selective MEK inhibitor PD 98059 can block expression of pERK and CDK2, and prevent versican G3 enhanced cell cycle entry and cell growth.

It is possible that signaling pathways associated with cell survival could also make a contribution to tumor invasion through a direct effect of versican on tumor cells. Glycogen synthase kinase-3β (GSK-3β), a serine/threonine protein kinase involved in glycogen metabolism and the EGFR-mediated signaling pathway, appears to play an important role in embryonic development and tumorigenesis [58],[59]. Over-expression of GSK-3β can induce apoptosis in tumor cells, whereas inactivation of GSK-3β through phosphorylation of the Serine 9 residue can reduce apoptosis and enhance cell survival [58],[60]. In the current study, we found that the activity of GSK-3 β (S9P) increases in versican G3 expressing cells, which is required for tumor cell survival and anti-apoptosis. Regulation of GSK-3β activity through both serine and tyrosine phosphoylation is a critical determinant of cell death or survival [61],[62]. Factors that promote cell survival, such as growth factors, activate EGFR/Akt which in turn phosphorylates GSK-3β at Serine 9, leading to inactivation of its kinase activity [62]. Selective EGFR AG inhibitor 1478 and ERK inhibitor PD 98059 prevent G3 induced phosphorylation of GSK-3β at Ser-9, leading to activation of GSK-3β activity, which is related to cell apoptosis.

Consistent with studies in vitro, in vivo experiments demonstrated that versican G3 enhanced the spontaneous metastasis of tumors from the mammary gland to distant organs including bone and contributed towards a more aggressive phenotype. G3's effect on in vivo local tumor growth was associated with changes in EGFR signaling, and p-ERK expression levels were observed to be more than two-fold greater in primary tumors of G3 treated mice as compared with those of the vector control group. To our knowledge, our study provides the first direct in vivo evidence that tumor specific expression of versican G3 domain, EGFR and pERK contributes to the spontaneous metastasis of mammary tumors from the fat pad to systemic distant organs. A more aggressive weight loss and lung metastasis pattern was observed in the G3 treated group when compared to the control group. Most importantly, we report in the present article that expression of the versican G3 domain in a mammary tumor cell line (66c14) that does not normally metastasize to bone is sufficient to promote their spontaneous metastasis to this tissue site. Whether this is predominantly an effect of G3 or of tumorgenicity in the time-course of metastatic spread warrants ongoing study although in vitro chemotactic motility assays did support enhanced G3 induced cell migration towards bone. Of interest would include evaluating factors (eg. alphavbeta3 integrin) that may promote chemotactic/haptotactic migration towards bone [63]. Versican expression may be important during the process of tumor bony invasion and subsequent remodeling of bone that leads to osteolysis with a resultant loss in mature organized bony micro-architecture [47]. Previous research has shown that the interaction of beta1-integrin with the C-terminal domain of PG-M/versican activates focal adhesion kinase enhancing integrin expression and promoting cell adhesion [15]. Versican G3 has been shown to interact with beta1-integrin in other cancer cell types [63],[64]. The increasing knowledge of several beta3 integrin-expressing cell populations, including osteolasts in breast cancer tumor progression, suggests that versican-integrin mediated interactions may be important in bony metastatic spread [63],[64].

To summarize, we have found that expression of versican G3 promoted breast cancer cell growth and metastasis through up-regulating active EGFR expression and activation of the EGFR-mediated pathway. Versican G3 domain appreciably increased breast cancer cell attachment, proliferation, and migration in vitro. G3 promoted tumor growth and systemic metastasis in vivo. Blockade of EGFR with AG1478 or blockade or ERK with PD 98059 inhibited versican G3 effects on cell proliferation. Blockade of EGFR also inhibited G3 effects on tumor cell chemotactic migration to bone stromal cells; while inhibition of EGFR and ERK did not significantly influence G3's effect on cell attachment. Although we do not know whether the high expression of EGFR signal is promoted by versican or activitated in association with other molecular determinants, understanding the signaling cascade is important towards the mechanisms of action in factors that influence tumor invasiveness.

## Supporting Information

Figure S1 Expression of Versican V1 isoform in of the 4 mouse breast cancer cell lines in RT-PCR.
(0.07 MB PDF)

Figure S2 Blockade of EGFR with AG 1478, or treating the cells with selective MEK inhibitor PD 98059 did not influence G3-induced cell attachment during the time period evaluated. (a) G3- and vector-transfected 66c14 cells (2×105) were inoculated in 6-well culture dishes in DMEM containing 2.5% FBS without or with AG 1478 (0.5, 2.0, and 5.0 µM) for 2 hr. (b) The G3- and vector-transfected 66c14 cells (2×105) were inoculated in 6-well culture dishes in DMEM containing 2.5% FBS with selective MEK inhibitor PD 98059 (20, 50, and 100 µM) for 2 hr. (All groups compared with G3 transfected 66c14 cells cultured in DMEM containing 2.5% FBS, n = 9, * p<0.05, ** p<0.01, analyzed with t-test).
(0.08 MB PDF)

Figure S3 Expression of G3 enhances cell proliferation and migration in human breast cancer cell lines. (a) 2×104 G3- and vector-transfected MT-1, MDA-MB-231, and MDA-MB-468 cells were inoculated in 6-well culture dishes with 10% FBS/DMEM and cultured for 3 days. More G3-transfected cells grew in these cell lines as compared with the control group. (All groups compared with vector control cells, n = 6, * p<0.05, ** p<0.01, analyzed with t-test). (b) The G3- and vector-transfected MDA-MB-468 cells (2×105) were inoculated in 6-well culture dishes and cultured for 12 h. Monolayer G3- and vector-transfected cells were wounded by a sterile pipette tip to create a 1-mm cell-free path, washed with PBS, and then cultured in 10% FBS/DMEM medium for 3 days. Pictures were taken under the light microscopy. The distances between the wounding centre and the front of the migrating cells (vertical axis) were measured for statistical analysis. (All groups compared with vector control cells, n = 10, * p<0.05, ** p<0.01, analyzed with t-test).
(0.08 MB PDF)

Figure S4 Typical flow cytometry plots showing that versican G3 domain promotes cell cycle entry, while AG 1478 prevented G3 enhanced S, G2 and M cell cycle status.
(0.48 MB PDF)

Figure S5 Typical flow cytometry plots showing that versican G3 domain promotes cell cycle entry, while PD 98059 prevented G3 enhanced S, G2 and M cell cycle status.
(0.49 MB PDF)
