# Supplementary figures and images for: Versican G3 Promotes Mouse Mammary Tumor Cell Growth, Migration, and Metastasis by Influencing EGF Receptor Signaling
Source: PLoS One. 2010 Nov 5;5(11):e13828. doi: 10.1371/journal.pone.0013828 (PMC2974650; doi:10.1371/journal.pone.0013828)

**a**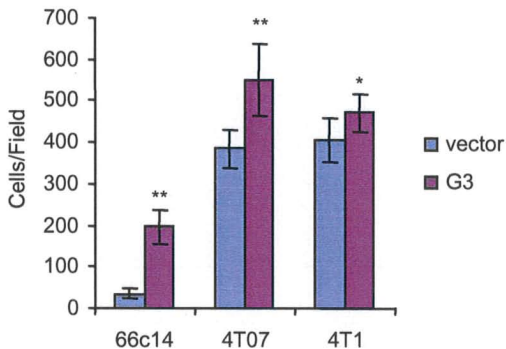**b**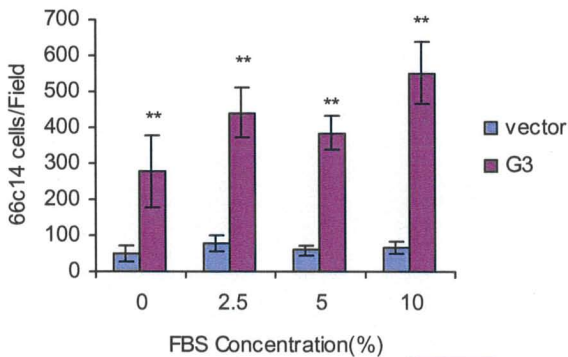**Fig 2**

Supplement: Figure S1 — Expression of Versican V1 isoform in of the 4 mouse breast cancer cell lines in RT-PCR. (0.07 MB PDF) [file pone.0013828.s001.pdf]

**a**

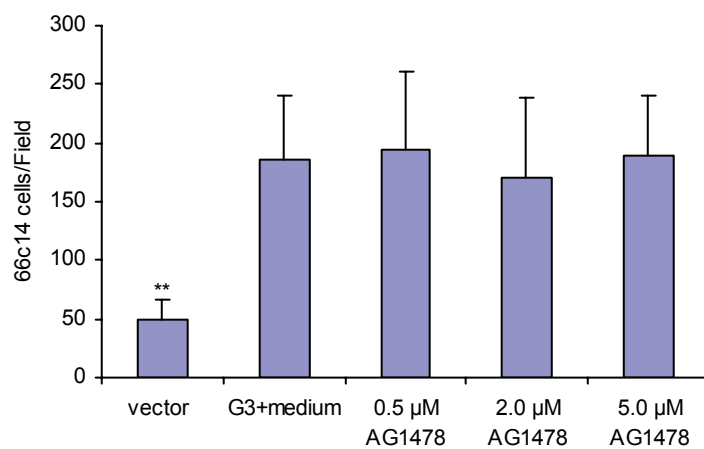

**b**

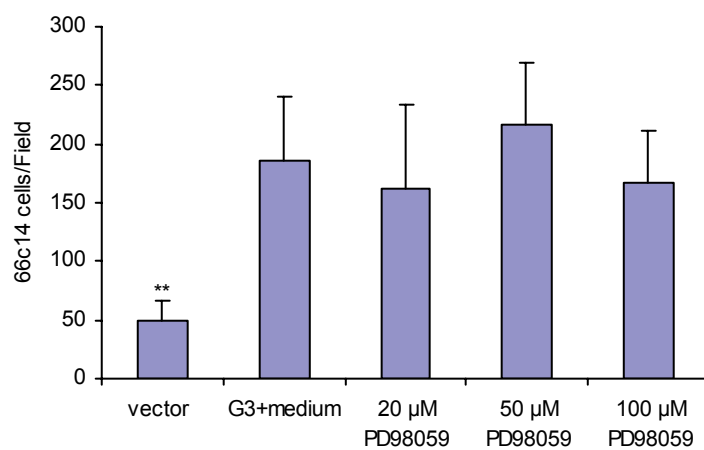

**Fig S2**

Supplement: Figure S2 — Blockade of EGFR with AG 1478, or treating the cells with selective MEK inhibitor PD 98059 did not influence G3-induced cell attachment during the time period evaluated. (a) G3- and vector-transfected 66c14 cells (2×10^5^) were inoculated in 6-well culture dishes in DMEM containing 2.5% FBS without or with AG 1478 (0.5, 2.0, and 5.0 µM) for 2 hr. (b) The G3- and vector-transfected 66c14 cells (2×10^5^) were inoculated in 6-well culture dishes in DMEM containing 2.5% FBS with selective MEK inhibitor PD 98059 (20, 50, and 100 µM) for 2 hr. (All groups compared with G3 transfected 66c14 cells cultured in DMEM containing 2.5% FBS, n = 9, * p<0.05, ** p<0.01, analyzed with t-test). (0.08 MB PDF) [file pone.0013828.s002.pdf]

**a**

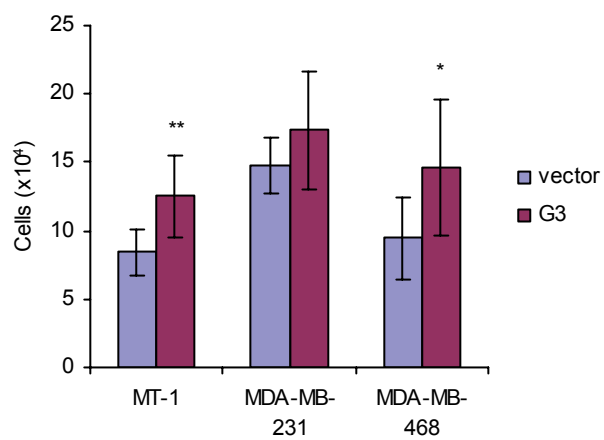

**b**

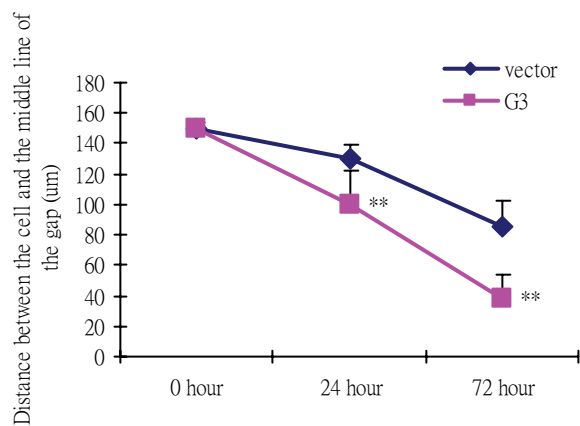

**Fig S3**

Supplement: Figure S3 — Expression of G3 enhances cell proliferation and migration in human breast cancer cell lines. (a) 2×10^4^ G3- and vector-transfected MT-1, MDA-MB-231, and MDA-MB-468 cells were inoculated in 6-well culture dishes with 10% FBS/DMEM and cultured for 3 days. More G3-transfected cells grew in these cell lines as compared with the control group. (All groups compared with vector control cells, n = 6, * p<0.05, ** p<0.01, analyzed with t-test). (b) The G3- and vector-transfected MDA-MB-468 cells (2×10^5^) were inoculated in 6-well culture dishes and cultured for 12 h. Monolayer G3- and vector-transfected cells were wounded by a sterile pipette tip to create a 1-mm cell-free path, washed with PBS, and then cultured in 10% FBS/DMEM medium for 3 days. Pictures were taken under the light microscopy. The distances between the wounding centre and the front of the migrating cells (vertical axis) were measured for statistical analysis. (All groups compared with vector control cells, n = 10, * p<0.05, ** p<0.01, analyzed with t-test). (0.08 MB PDF) [file pone.0013828.s003.pdf]
