# Supplementary material for: Versican G3 Promotes Mouse Mammary Tumor Cell Growth, Migration, and Metastasis by Influencing EGF Receptor Signaling
Source: PLoS One. 2010 Nov 5;5(11):e13828. doi: 10.1371/journal.pone.0013828 (PMC2974650; doi:10.1371/journal.pone.0013828)
Supplement: Figure S4 — Typical flow cytometry plots showing that versican G3 domain promotes cell cycle entry, while AG 1478 prevented G3 enhanced S, G2 and M cell cycle status. (0.48 MB PDF) [file pone.0013828.s004.pdf]

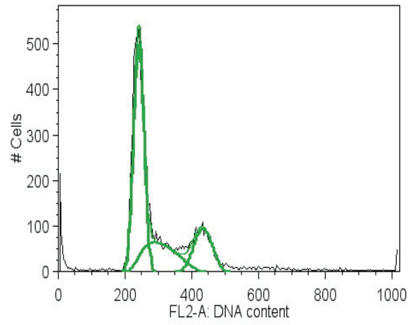

Dean/Jett/Fox: %G1=64.5, %S=21.7, %G2=16.7.

Vector in 0 μM AG 1478

Event Count: 10000

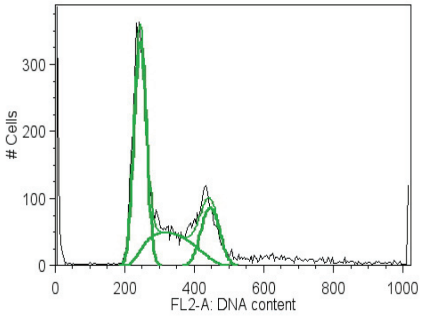

Dean/Jett/Fox: %G1=41.2, %S=38.6, %G2=24.5.

G3 in 0 μM AG 1478

Event Count: 10000

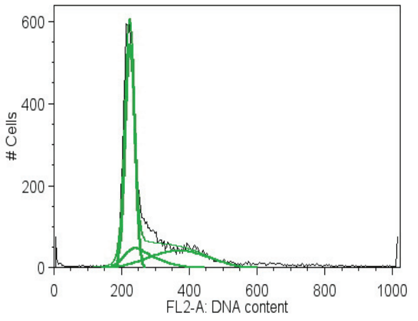

Dean/Jett/Fox: %G1=61.5, %S=16.3, %G2=24.1.

Vector in 0.5 μM AG 1478

Event Count: 10000

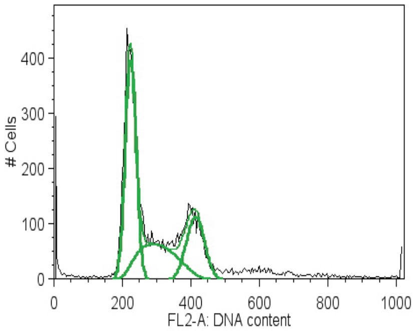

Dean/Jett/Fox: %G1=49.3, %S=29.3, %G2=21.8.

G3 in 0.5 μM AG 1478

Event Count: 10000

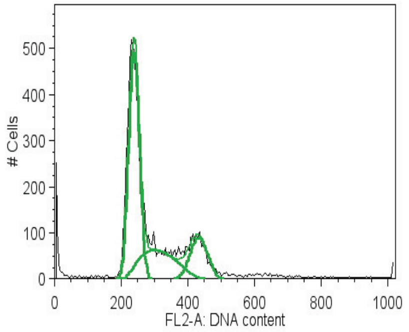

Dean/Jett/Fox: %G1=57.5, %S=21.7, %G2=17.8.

Vector in 2.0 μM AG 1478

Event Count: 10000

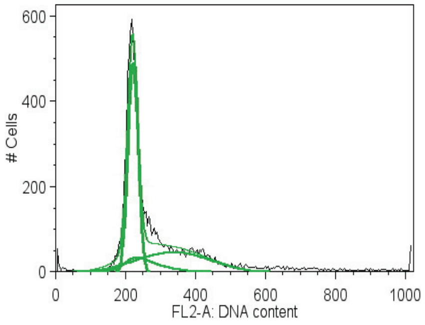

Dean/Jett/Fox: %G1=52.3, %S=13.7, %G2=25.7.

G3 in 2.0 μM AG 1478

Event Count: 10000

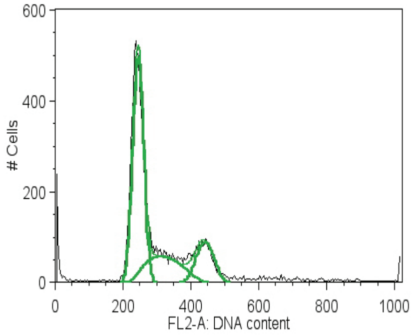

Dean/Jett/Fox: %G1=56.6, %S=23.5, %G2=15.6.

vector in 5.0 μM AG 1478

Event Count: 10000

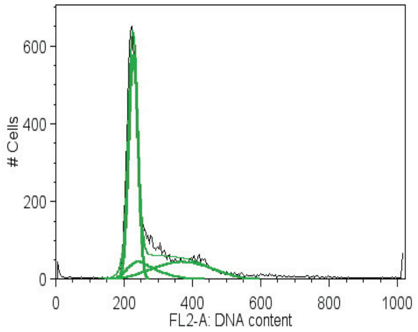

Dean/Jett/Fox: %G1=55.7, %S=15.7, %G2=20.8.

G3 in 5.0 μM AG 1478

Event Count: 10000

Fig S4
