# Supplementary material for: Versican G3 Promotes Mouse Mammary Tumor Cell Growth, Migration, and Metastasis by Influencing EGF Receptor Signaling
Source: PLoS One. 2010 Nov 5;5(11):e13828. doi: 10.1371/journal.pone.0013828 (PMC2974650; doi:10.1371/journal.pone.0013828)
Supplement: Figure S5 — Typical flow cytometry plots showing that versican G3 domain promotes cell cycle entry, while PD 98059 prevented G3 enhanced S, G2 and M cell cycle status. (0.49 MB PDF) [file pone.0013828.s005.pdf]

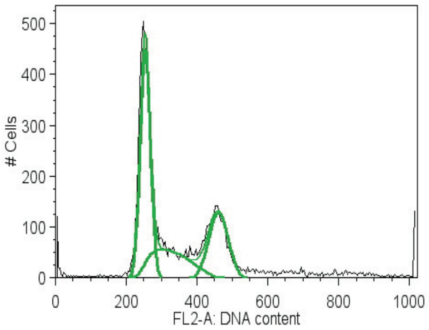

Dean/Jett/Fox: %G1=54.3, %S=18.7, %G2=19.9.
Vector in 0 μM PD 98059
Event Count: 10000

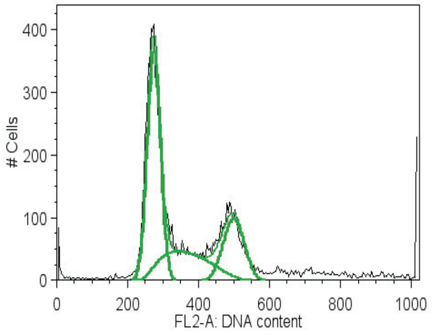

Dean/Jett/Fox: %G1=37.8, %S=29.6, %G2=27.1.
G3 in 0 μM PD 98059
Event Count: 10000

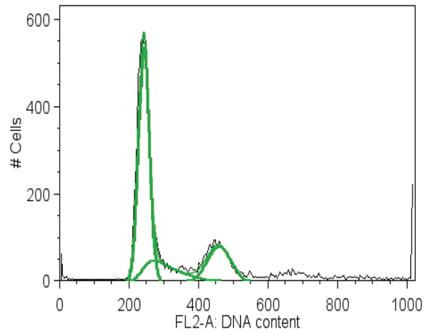

Dean/Jett/Fox: %G1=57.6, %S=17.3, %G2=19.2.
vector in 20 μM PD 98059
Event Count: 10000

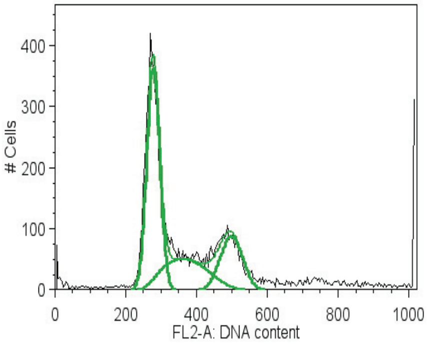

Dean/Jett/Fox: %G1=44.9, %S=27.3, %G2=22.8.
G3 in 20 μM PD 98059
Event Count: 10000

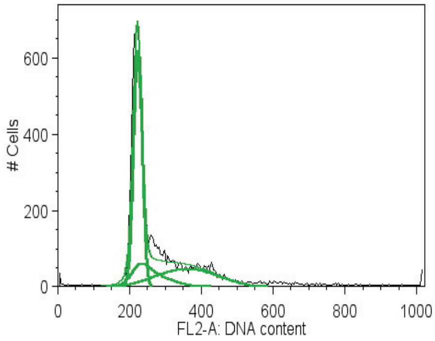

Dean/Jett/Fox: %G1=62.7, %S=11.4, %G2=24.2.
vector in 50 μM PD 98059
Event Count: 10000

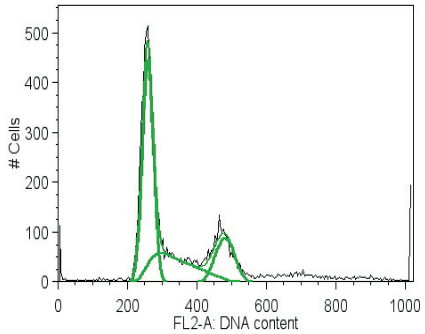

Dean/Jett/Fox: %G1=59, %S=21.7, %G2=15.7.
G3 in 50 μM PD 98059
Event Count: 10000

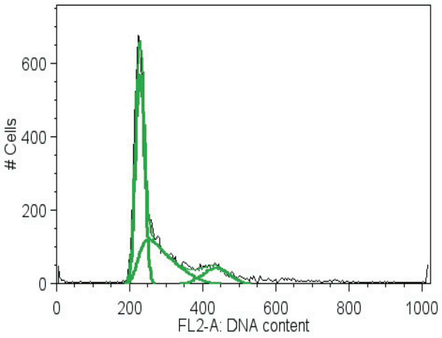

Dean/Jett/Fox: %G1=61.3, %S=22.6, %G2=12.2.
vector in 100 μM PD 98059
Event Count: 10000

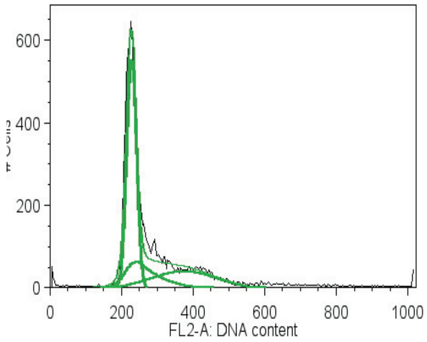

Dean/Jett/Fox: %G1=59.5, %S=15.8, %G2=22.1.
G3 in 100 μM PD 98059
Event Count: 10000

Fig S5
